# Meeting of the Missouri State Medical Association

**Published:** 1897-04

**Authors:** 


### Society Notes.

### Meeting of the Missouri State Medical Association, May 18, 19 and 20.

Present prospects are that the meeting of the Missouri State Association this year is going to prove very satisfactory. The committees have all gotten to work early which is a good indication. The committee on scientific communications is already in receipt of titles in numbers and character sufficient to insure the programme scientifically attractive. The executive committee are enabled to announce the following programme, the details of which only remain to be completed. The association will meet in St. Louis, May 18, 19 and 20. All the first, the second and the third day until noon will be devoted to the scientific programme. On the evening of the first day the association will as a body attend a session of the Illinois Society in East St. Louis. On the evening of the second day the Illinois Society will attend as a body a session of the Missouri Associaciation, after which there will be a banquet and reception. On the third day both bodies will adjourn and join in a steamboat excursion on the river.

---

